# Homeostatic signals, including IL-7 and self-MHC recognition, induce the development of peripheral helper T cells, which are enriched in the joints of rheumatoid arthritis

**DOI:** 10.1016/j.jtauto.2024.100258

**Published:** 2024-10-30

**Authors:** Ryosuke Tsurui, Hisakata Yamada, Takahiro Natori, Motoki Yoshimura, Yukio Akasaki, Shinya Kawahara, Hiroaki Niiro, Yuya Kunisaki, Yasuharu Nakashima

**Affiliations:** a Department of Orthopaedic Surgery, Graduate School of Medical Sciences, Kyushu University, Fukuoka, Japan; b Department of Clinical Immunology, Graduate School of Medical Sciences, Kyushu University, Fukuoka, Japan; c Department of Medicine and Biosystemic Science, Graduate School of Medical Sciences, Kyushu University, Fukuoka, Japan; d Department of Medical Education, Graduate School of Medical Sciences, Kyushu University, Fukuoka, Japan; e Department of Clinical Chemistry and Laboratory Medicine, Graduate School of Medical Sciences, Kyushu University, Fukuoka, Japan

**Keywords:** Rheumatoid arthritis, Peripheral helper T cells, Interleukin-7

## Abstract

**Objective:**

Dysregulated T cell homeostasis has long been implicated in the pathogenesis of rheumatoid arthritis (RA), in the joint of which peripheral helper T (Tph) cells accumulate and form ectopic lymphoid organs. We examined whether homeostatic signals are involved in the development of Tph cells.

**Methods:**

Human peripheral blood mononuclear cells were cultured with IL-7, the critical cytokine for T cell homeostasis. Development of Tph-like cells was assessed by flow cytometry, gene expression, and functional analysis. Chemotaxis of the Tph-like cells to RA synovial fluid (RASF) and the effect of RASF on the development of Tph-like cells was examined.

**Results:**

PD-1^high^CXCR5^-^ Tph-like cells developed from human peripheral blood CD4 T cells after proliferation in response to IL-7. Signals from self-MHC recognition and CD28 co-stimulation were also involved. The IL-7-induced Tph-like (IL-7-Tph) cells produced CXCL13 and IL-21 and helped B cells produce IgG. Comprehensive gene expression analysis further supported the similarity with Tph cells in RA joint. IL-7-Tph cells exhibited chemotaxis toward synovial fluid from RA patients (RASF), and RASF promoted the development of IL-7-Tph cells, which were also induced from CD4 T cells residing in non-inflamed joints.

**Conclusions:**

Our results demonstrate an antigen-nonspecific developmental pathway of Tph cells triggered by homeostatic signals and promoted by the local environment of RA, which accounts for the accumulation of Tph cells in inflamed joints.

## Highlights

• In vitro culture of human CD4 T cells with IL-7 induces Tph-like (IL-7-Tph) cells.
• IL-7-Tph cells are phenotypically and functionally similar to Tph cells in RA joint.
• Signals from self-MHC and co-stimulation are also involved in IL-7-Tph development.
• Synovial fluid from RA patients recruits IL-7-Tph and promotes their development.

## 1 Introduction

Rheumatoid arthritis (RA) is a chronic inflammatory disease that primarily targets synovial joints and accompanies systemic abnormality in adaptive immunity. Thus, autoantibodies, such as rheumatoid factor (RF) and anti-citrullinated protein antibody (ACPA), are detected in the serum, which is implicated in the development of RA [1]. In addition, dysregulated T cell homeostasis resulting from premature immune senescence has long been known [2,3]. Although an importance of CD4 T cells in the pathogenesis of RA has been suggested by the genetic association with HLA-DR and the clinical effectiveness of co-stimulation blockade with CTLA4-Ig [4,5], the relationship between the systemic T cell abnormality and the joint pathology remains unclear.

Ectopic lymphoid organs (ELOs) are occasionally detected in chronically inflamed tissues, including synovium of RA. ELO formation in RA synovium is associated with the severity of arthritis and is involved in local antibody production including ACPA [6,7], Peripheral helper T (Tph) cells, which were first identified in the joint of RA, are implicated in the formation of ELOs [8]. Tph cells are abundantly found in the joint of seropositive RA but are hardly detected in seronegative arthritis as well as non-inflammatory joints [8,9]. Tph cells resemble follicular helper T (Tfh) cells, which reside in the follicles of secondary lymphoid organs, in that both express PD-1, secrete IL-21 and CXCL13, and help B cells for antibody production, but only Tfh cells express CXCR5 [8]. The expression pattern of transcription factors also differs between Tph and Tfh cells: although Maf is expressed by both, expression of Blimp 1 and Bcl 6 is mutually exclusive [10]. Different ability of helping naïve B cells by Tph and Tfh cells was also reported [11]. Tph cells are equipped with multiple Th1-like effector cytokines, suggesting their role in driving local inflammation besides helping B cells [12]. Tph cells are found in various inflammatory disorders, including Celiac disease, systemic lupus erythematosus, and Sjogren's syndrome [[13], [14], [15]]. PD-1^high^CXCR5^−^CD4 T cells resembling Tph cells are detected in the peripheral blood, circulating Tph (cTph) cells, and are increased in inflammatory conditions, though at much lower frequency than tissue Tph cells [8,11,13,16].

Elucidating the mechanism of Tph cell development would help understand the pathogenesis and develop treatment strategies of inflammatory disorders, including RA. Yoshitomi et al. reported induction of Tph-like, CXCL13-producing cells from human naïve CD4 T cells by adding TGF-β to anti-CD3 mAb-stimulation, which was promoted by blocking IL-2 [17]. They later identified Sox4 as the essential transcription factor for the development of CXCL13-producing cells [18]. However, it does not fully account for the development of Tph cells, as the CXCL13-producing cells did not produce IL-21. Expression of Sox4 in Tph cells in RA joint has not been demonstrated. Based on the assumption that Tph cells are clonally expanded autoreactive cells, there have been studies analyzing the clonality of Tph cells in RA joints. Argyriou et al. showed by single-cell RNA sequencing (RNAseq) analysis that the frequency of expanded clones was highest in Tph cells among CD4 T cells [10]. However, Tph cells in RA joints are still highly polyclonal and sometimes make up the majority of CD4 T cells [9,10,12,19], which may not simply be attributed to the conventional antigen-driven clonal expansion and suggests the presence of antigen-nonspecific developmental pathway.

T cells can be activated and proliferate in an antigen-nonspecific manner, bystander activation, which is well known in the context of infection [20], Memory phenotype T cells respond to proinflammatory cytokines, including IL-12 and IL-15, and contribute to host defense. Naïve T cells also proliferate in an antigen-nonspecific manner in lymphopenic conditions, which is called lymphopenia-induced proliferation (LIP) and is mediated by IL-7 signaling [21]. LIP is a mechanism maintaining peripheral T cell pool and is also called "homeostatic" proliferation. However, naive T cells that have undergone LIP acquire phenotype and functions of memory T cells, in which low-affinity recognition of self-MHC molecules is involved [22,23]. Memory phenotype T cells also exhibit LIP [24,25]. Interestingly, it was demonstrated in mice that Tph-like, PD-1^+^CXCR5^-^ cells with B cell-helping functions developed from naïve CD4 T cells after LIP, suggesting alternative pathway of Tph development [26]. PD-1^+^CXCR5^-^ CD4 T cells producing IL-21 were detected in ELO in the kidney of aged mice [27]. These findings suggest similar mechanisms operating in the development of human Tph cells in individuals with disturbed T cell homeostasis and ELO formation, such as RA. In the present study, we examined whether homeostatic signals induce antigen-nonspecific development of Tph cells from human CD4 T cells and investigated its relevance in the context of RA.

## 2 Materials and methods

### 2.1 Patients and samples

Blood samples were obtained from 12 healthy volunteers and 6 RA patients. Synovial fluid (SF) samples were obtained from 18 RA patients by arthrocentesis, while synovial membrane (SM) samples were obtained from 3 osteoarthritis (OA) patients at the time of arthroplasty. All RA patients met the 1987 ACR or the 2010 ACR/EULAR classification criteria at the time of diagnosis. Demographics of RA and OA patients are shown in Supplemental Table 1. This study complies with the Declaration of Helsinki, and the study protocol was approved by the Kyushu University School of Medicine Human Research Ethics Regional Committee (2020-333). Signed informed consent was obtained from all subjects before participation in the study.

### 2.2 Preparation of mononuclear cells

Peripheral blood mononuclear cells (PBMCs) were isolated by using Ficoll-Paque Plus (GE Healthcare Bio-Science, Uppsala, Sweden). For the isolation of SFMCs, SF samples were treated with 20 μg/ml hyaluronidase (Sigma-Aldrich, St. Louis, MO) for 30 min at 37 °C, and filtered through a 70 μm cell strainer (Corning, NY). SFMC were isolated using Ficoll-Paque Plus. The supernatant was stored at −80 °C until use. SM samples were cut into 1–2 mm pieces and digested with 2 mg/ml collagenase (Wako, Osaka, Japan) and 10 μg/ml DNase (Roche, Basel, Switzerland) in culture medium (RPMI 1640 with 10 % fetal bovine serum, 500 units/ml penicillin, 500 μg/ml streptomycin, and 0.1 μM 2-mercaptoethanol) at 37 °C in a shaking incubator for 30 min. The digested samples were filtered through a 70 μm cell strainer and SMMC were isolated using Ficoll-Paque Plus.

### 2.3 Cell culture

PBMCs were incubated in the culture medium with recombinant human IL-7 (Thermo Fisher, Waltham, MA) up to 14 days. Concentration of IL-7 was 50 ng/ml unless specified. To assess proliferation, cells were stained with CellTrace Violet (CTV; Thermo Fisher) prior to culture. Culture plates were precoated with 1 μg/ml anti-CD3 (clone OKT3, Thermo Fisher) and 2 μg/ml anti-CD28 (clone CD28.2, BD Biosciences, San Diego, CA) mAb to induce TCR-stimulated T cells in PBMC. For the blocking experiments, 10 μg/ml of neutralizing anti-HLA-DR antibody (clone L243; BioLegend, San Deigo, LA) or CTLA4-Ig (abatacept; Bristol Myers Squibb, NY) was added to the culture.

### 2.4 Flow cytometric analysis

Fluorochrome-conjugated mAbs used for flow cytometric analysis were listed in Supplemental Table 2. Optimally diluted mAb were added to the cell suspension in PBS containing 2 % fetal bovine serum for 20 min at 4 °C. Propidium iodide (2 μg/ml) was added to the cell suspension just before run on a FACSVerse flow cytometer (BD Biosciences). The data were analyzed by using FlowLogic Software (Miltenyi Biotec, Bergosch Gladbach, Germany). For intracellular staining, cells were stimulated with 50 ng/ml PMA (Sigma-Aldrich) and 1 μg/ml ionomycin (Sigma-Aldrich) or left unstimulated in the presence of 10 μg/ml brefeldin A (Sigma-Aldrich) for 4 h. After staining surface molecules, intracellular staining of cytokines was performed by using BD Cytofix/Cytoperm solution and BD Perm/Wash solution following the manufacturer's instruction.

### 2.5 Cell separation and sorting

For magnetic depletion of PD-1 or CXCR5-expressing cells, anti-PE MACS microbeads (Miltenyi Biotec) and PE-streptavidin were used in combination with biotin-conjugated anti-PD-1 or anti-CXCR5 mAbs, respectively. Negative selection of CD4 T cells were performed by using CD4 T Cell isolation kit (Miltenyi Biotec). Flow cytometric cell sorting of the cultured PD-1^high^CTV^low^ CD4 T cells, CD45RA^+^RO^−^ naive and CD45RA^−^RO^+^ memory CD4 T cells in PB, CD19^+^CD27^+^ memory B cells in PB, PD-1^high^CXCR5^-^ CD4 T (RA-Tph) cells in RASF was performed on a FACSAria Fusion cell sorter (BD Biosciences).

### 2.6 T and B cell co-culture

Co-culture experiments were performed as described [28]. In brief, sorted PD-1^high^CTV^low^ CD4 T cells in IL-7-stimulated PBMCs or naïve CD4 T cells and CD19^+^CD27^+^ memory B cells from PBMCs of the same donor were cultured at a ratio of 1:5 in the presence of staphylococcal enterotoxin B (1 mg/mL) and LPS (5 mg/mL) for 7 days. Total IgG in the supernatant was measured by an ELISA kit (Thermo Fisher). Cultured cells were examined for the development of CD27^+^CD38^+^ plasmablasts.

### 2.7 Bulk RNAseq analysis

Total RNA was purified using the SMART-seq HT Plus kit (Takara Bio, Shiga, Japan). Barcoded cDNA libraries were generated by the Nextera XT DNA Library Preparation kit (Illumina) and sequenced with a 150bp × 2 paired-end protocol on the NovaSeq 6000 System (Illumina) at Rhelixa (Tokyo, Japan).

The quality of the raw paired-end sequence reads was assessed with FastQC version 0.11.7. Low quality (<20) bases and adapter sequences were trimmed by Trimmomatic software version 0.38. The trimmed reads were aligned to the reference genome using RNA-seq aligner HISAT2 version 2.1.0. The abundance of uniquely mapped reads was estimated by featureCounts version 1.6.3, and iDEP.2.01 was used for hierarchical clustering with a heatmap, identification of differentially expressed genes (DEGs), pathway analysis, and principal component analysis (PCA). DEGs were extracted with false discovery rate (FDR) cutoff of 0.1 and min-fold change of 2. Gene set enrichment analysis (GSEA) was performed by using the gene sets in Kyoto Encyclopedia of Genes and Genomes. Pathway significance FDR cutoff was set at 0.1.

### 2.8 Quantitative real-time RT-PCR analysis

Total RNA was purified using FastGene™ RNA Premium Kit (NIPPON Genetics, Tokyo, Japan). cDNA was synthesized using PrimeScript RT reagent kit (Takara Bio, Kusatsu, Japan). Quantitative real-time RT-PCR was performed using CFX Maestro (BIORAD, Hercules, CA) and STBR Premix EX *Taq*II (Takara Bio). Data are presented as either 2-ΔCT, where ΔCT = CT for GAPDH minus CT for genes of interest, or fold-change, where ΔCT for the control = 1. The following primer pairs were used:

GAPDH-F: 5′-GGTGAAGGTCGGAGTCAACGGA-3′

GAPDH-R: 5′-GAGGGATCTCGCTCCTGGAAGA-3′

MAF-F: 5′-CACCCTGCTCGAGTTTGTG-3′

MAF-R: 5′-CATGAGCCAGACACCCATT -3′

PRDM1-F: 5′-AACTTCTTGTGTGGTATTGTCGG -3′

PRDM1-R: 5′-TCTCAGTGCTCGGTTGCTTT-3′

BCL6-F: 5′-GTTTCCGGCACCTTCAGACT-3′

BCL6-R: 5′-CTGGCTTTTGTGACGGAAAT-3′

### 2.9 Chemotaxis assay

Chemotaxis of T cells was investigated by a Boyden chamber assay using 24-well chemotaxis plates with 3 μm pore size of polycarbonate filters (Corning, Corning, NY), as previously reported [12]. Briefly, the lower wells were filled with 600 μl of assay buffer (RPMI 1640 with 0.1 % BSA) with or without 20 % of RASF. Cultured PBMCs were placed in upper wells at 2.0 × 10^5^ per 200 μl of assay buffer and incubated for 4 h. Cells that migrated into the lower wells were collected and analyzed by flow cytometry.

### 2.10 Cytometric Bead Array assay

The levels of cytokines and growth factors in the SF were quantified using Cytometric Bead Array (CBA™, BD Biosciences), according to the manufacturer's instructions. Each sample was incubated with a set of beads coated with antibodies specific to the targeted cytokines or growth factors. After incubation, the beads were washed and then treated with a secondary antibody specific to the cytokines. A standard curve was established for each cytokine. Fluorescence measurements were acquired using a FACSverse flow cytometer, and the data were analyzed with CBA analysis software (BD Biosciences).

### 2.11 Statistics

Shapiro-Wilk test was used to assess normal distribution of the data. For normally distributed data, two-tailed Student's t-test was applied in comparing two groups. In comparing more than two groups, one-way analysis of variance (ANOVA) was performed, followed by Tukey's post hoc test. For non-normally distributed data, the Kruskal-Wallis test was used, with Dunn's post hoc test for more than two groups. All statistical analyses were conducted using Prism 9 software (GraphPad Software, La Jolla, CA). A p-value of <0.05 was considered statistically significant.

## 3 Results

### 3.1 IL-7 induces antigen-nonspecific development of PD-1^high^CXCR5^-^ Tph-like cells from human PB CD4 T cells

**Fig. 1:**
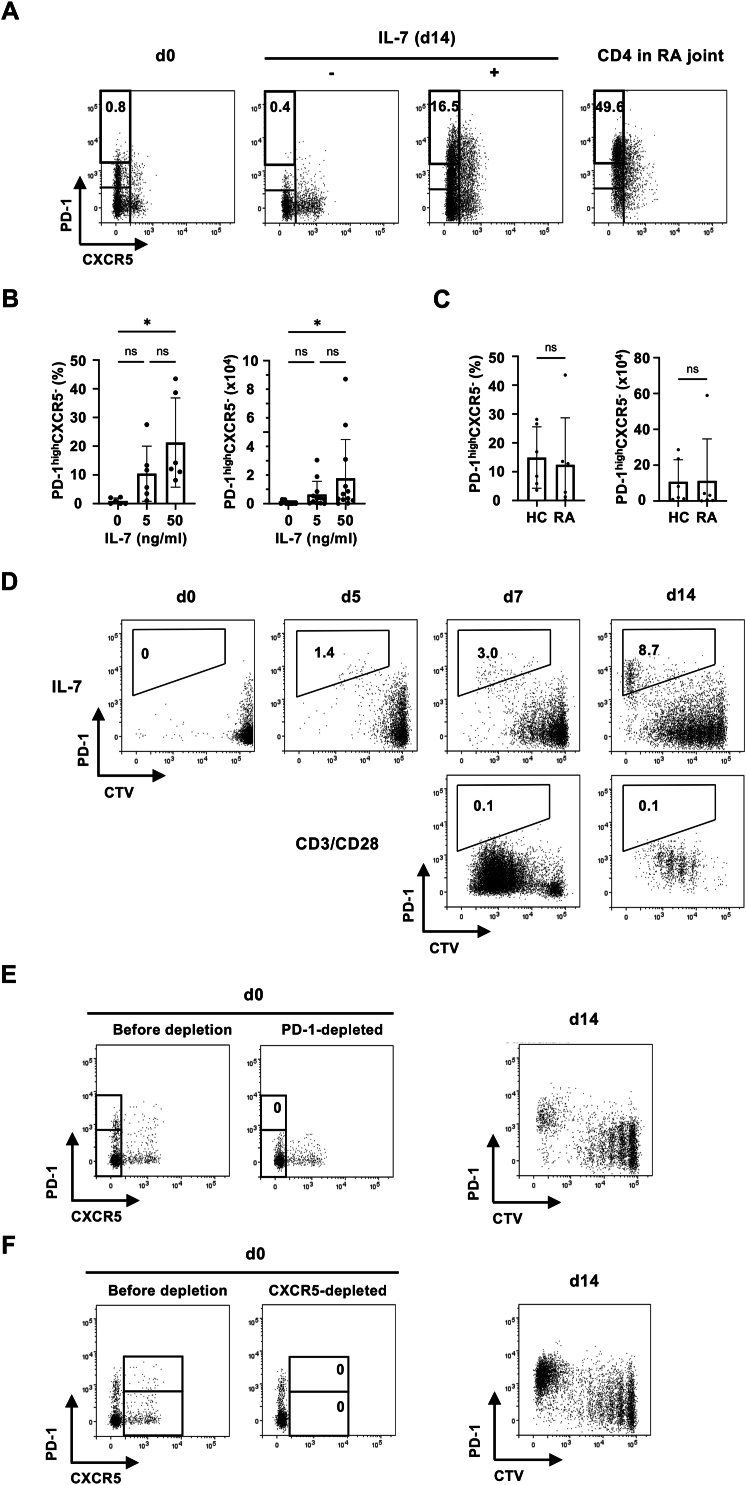
IL-7 induced the development of PD-1^high^CXCR5- Tph-like cells from human PB CD4 T cells. (A) PBMCs from healthy human subjects were cultured with or without 50 ng/ml of IL-7 for 14 days. Representative dot plots showing the expression of PD-1 and CXCR5 on CD4 T cells are shown. The right panel (CD4 in RA joint) indicates the staining pattern of CD4 T cells isolated from RA joint. (B) The frequency (left) and cell number in a culture well (right) of PD-1^high^CXCR5^-^ Tph-like cells induced with varying concentration of IL-7. (C) The frequency and number of PD-1^high^CXCR5^-^ Tph-like cells induced from PBMC of HC and RA are shown (n = 6). (D) CTV-labelled PBMCs were cultured either with IL-7 (upper panels) or in plate wells precoated with anti-CD3 and anti-CD28 mAbs (lower panels). Expression of PD-1 and CTV staining of CD4 T cells cultured for the indicated days is shown. Induction of PD-1^high^CXCR5^-^ Tph-like cells from PD-1-depleted (E) or CXCR5-depleted (F) PBMCs by IL-7. Left panels (d0) indicate the expression of PD-1 and CXCR5 on CD4 T cells before and after negative selection. ∗p < 0.05.

We first examined whether IL-7, the critical cytokine maintaining T cell homeostasis, induces the development of Tph cells from human CD4 T cells. PBMCs from healthy individuals were cultured in the presence or absence of IL-7 for two weeks. We found substantial portion of IL-7-stimulated CD4 T cells expressing high levels of PD-1(PD-1^high^) without CXCR5, comparable to the levels detected on CD4 T cells isolated from RA joints (Fig. 1A). A weak dose response to IL-7 was observed, thereafter we used 50 ng/ml of IL-7 in the following experiments (Fig. 1B). The PD-1^high^CXCR5^-^ Tph-like cells were also induced from PBMCs in RA patients (Fig. 1C). By staining PBMCs with CTV before culture, we verified the relationship between IL-7-induced CD4 T cell proliferation and expression of PD-1: high levels of PD-1 were expressed on cells exhibiting extensive cell division (Fig. 1D). Importantly, PD-1^high^CTV^low^ cells were not detected when T cells were stimulated with anti-CD3 and anti-CD28 mAbs, although they did show cell division, suggesting that the development of Tph-like cells in this model results from IL-7-induced proliferation. Although a portion of CD4 T cells in PB expressed intermediate levels of PD-1 (PD-1^int^) before culture, PD-1^high^ cells were not induced from PD-1^int^ cells, because depleting PD-1-expressing cells before culture did not prevent the appearance of PD-1^high^ cells (Fig. 1E). Depleting CXCR5^+^ cells before culture did not affect either, indicating that the Tph-like cells are not derived from cTfh cells (Fig. 1F).

### 3.2 Similarity of the IL-7-induced Tph-like cells and Tph cells in RA joint

**Fig. 2:**
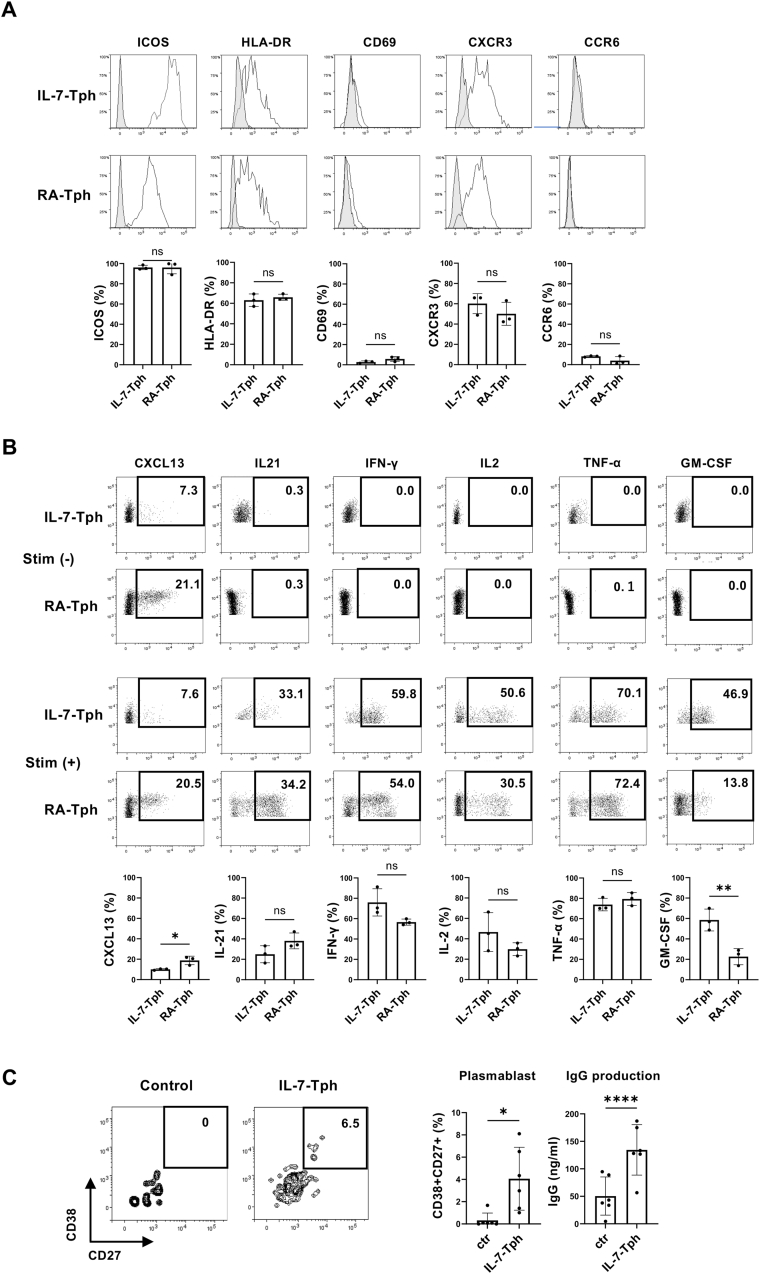
Phenotypical and functional similarities between the IL-7-induced Tph-like (IL-7-Tph) cells and Tph cells in RA joint (RA-Tph cells). (A) Expression of surface molecules (ICOS, CD69, HLA-DR, CXCR3, and CCR6) on IL-7-Tph (n = 3) and RA-Tph cells (n = 3) are compared in histograms. Gray histograms indicate the staining with isotype controls. Graphs indicate the mean percentage of positive cells in PD-1^high^ CD4 T cells (n = 3). (B) Representative dot plots of cytokine production by the IL-7-Tph and RA-Tph cells with (lower panels) or without (upper panels) stimulation with PMA and ionomycin are shown. Graphs indicate the mean percentage of cytokine positive cells in PD-1^high^ CD4 T cells (n = 3). (C) Induction of plasmablasts (CD27^+^ CD38^+^) and IgG production from memory B cells cocultured with IL-7-Tph cells(n = 6). Representative dot plots of CD38 and CD27 expression on B cells are shown in left panels. ∗p < 0.05, ∗∗p < 0.01, ∗∗∗∗p < 0.0001.

The IL-7-induced Tph-like (IL-7-Tph) cells expressed ICOS and HLA-DR, like Tph cells in RA joint (RA-Tph) (Fig. 2A). In line with the expression of CXCR3 without CCR6, Th1-related effector cytokines including IFN-γ and TNF-α were produced by both (Fig. 2A and B). IL-7-Tph and RA-Tph are also similar in the expression of IL-21 and CXCL13, the functional hallmark of Tph cells, although the expression level of CXCL13 was lower in IL-7-Tph cells than RA-Tph cells. Importantly, spontaneous CXCL13 production without stimulation was detected in both. Functional relevance of the cytokine expression was confirmed by in vitro coculture experiments, in which memory B cells cultured with the IL-7-Tph cells differentiated into plasmablasts and secrete IgG in the supernatant (Fig. 2C and D).

**Fig. 3:**
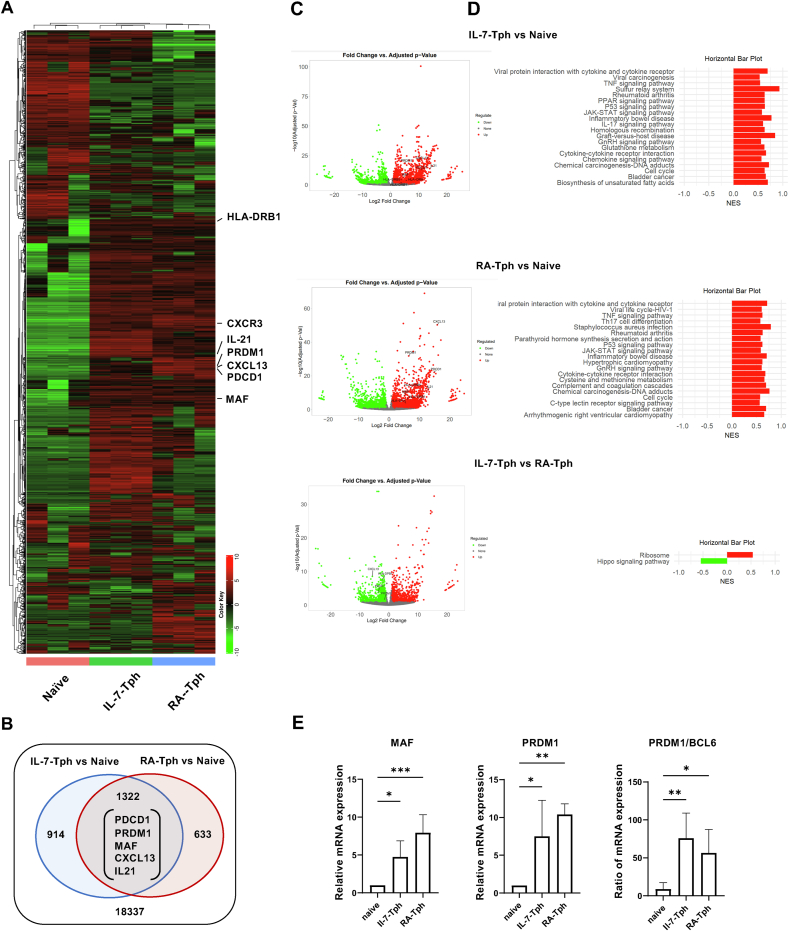
Gene expression analysis of the IL-7-Tph cells. (A) Heatmap showing hierarchical clustering by DEGs among sorted naïve CD4 T (n = 3), IL-7-Tph (n = 3), and RA-Tph cells (n = 3). (B) Venn diagrams showing the number of the upregulated genes overlapped in IL-7-Tph and RA-Tph cells compared to naive CD4 T cells. (C) Volcano plots showing DEGs between IL-7-Tph and naive CD4 (upper), RA-Tph and naive CD4 (middle), and IL-7-Tph and RA-Tph (lower) cells. (D) GSEA analysis showing the pathway of genes upregulated in (C). (E) Expression levels of *MAF*, *PRDM1*, and *PRDM1/BCL6* ratio in IL-7-Tph and RA-Tph cells by qRT-PCR analysis (n = 5). ∗p < 0.05, ∗∗p < 0.01, ∗∗∗p < 0.001.

To further verify the similarity between IL-7-Tph and RA-Tph cells, transcriptome analysis by bulk RNAseq was performed. Hierarchal clustering by DEGs showed similarity between IL-7-Tph and RA-Tph cells (Fig. 3A). There was a large overlap of upregulated genes, including *PDCD1*, *MAF, PRDM1*, *CXCL13*, and *IL21* (Fig. 3B and C). Notably, *SOX4* and *BCL6*, key transcription factors for the TCR-induced CXCL13-producing cells [18] and Tfh cells [29], respectively, were not upregulated in IL-7-Tph or RA-Tph cells. GSEA analysis showed more than half of upregulated pathways, including cytokine-cytokine receptor interaction, inflammatory bowel disease, and rheumatoid arthritis, common in IL-7-Tph and RA-Tph cells (Fig. 3D). Only two pathways differ between IL-7-Tph and RA-Tph cells. Expression level of the key molecules was confirmed by qRT-PCR analysis (Fig. 3E): IL-7-Tph and RA-Tph cells expressed increased levels of *MAF*, *PRDM1, and BCL6/PRDM1 ratio*. Thus, IL-7-Tph and RA-Tph cells are functionally similar.

### 3.3 Signal requirements and precursors of IL-7-Tph cells

**Fig. 4:**
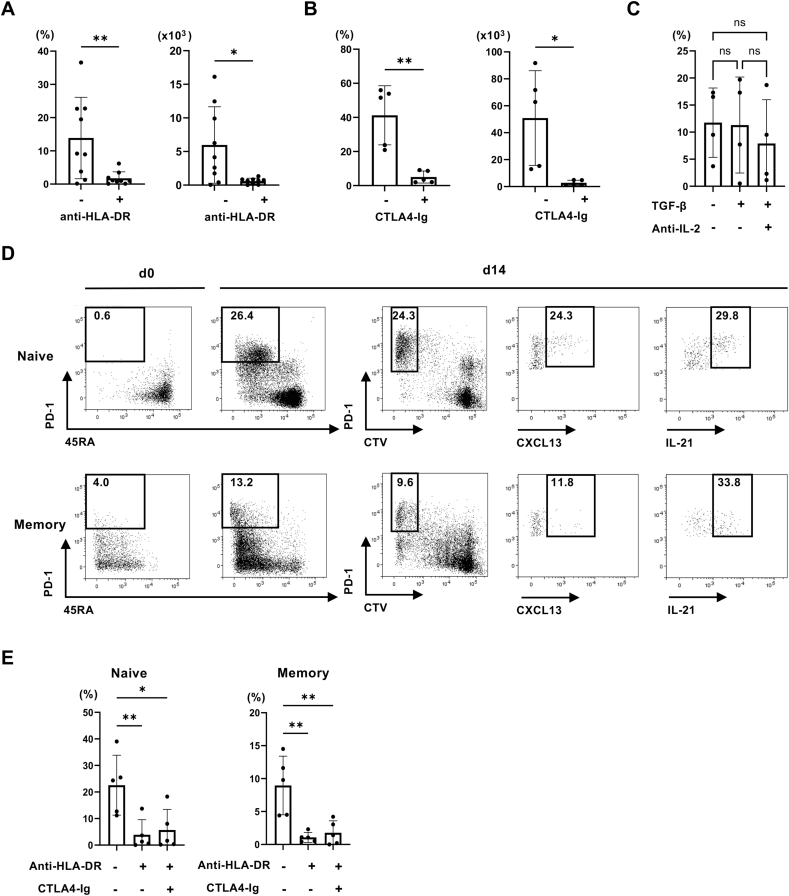
Signalling requirements for the development of IL-7-Tph cells. The frequency (left) and cell number in a culture well (right) of PD-1^high^ CTV^low^ cells after culturing PBMC with IL-7 in the presence or absence of anti-DR mAb (A) or CTLA-4 Ig (B) is shown (n = 9). (C) Effect of adding anti-IL-2 mAb and TGF-β was examined (n = 4). (D) Representative dot plots showing the induction of IL-7-Tph cells from either naïve (upper panels) or memory (lower panels) CD4 T cells as depicted by the expression of CD45RA and PD-1 (d0 and d14 left). Expression of CXCL13 and IL-21 in PD-1^high^ CTV^low^ cells after stimulation was shown in the right panels. (E) The effect of adding anti-DR mAb or CTLA-4 Ig on the induction of IL-7-Tph cells from naive (left) or memory (right) CD4 T cells (n = 5). ∗p < 0.05, ∗∗p < 0.01.

It is of interest to determine whether IL-7-Tph developed from naïve or memory CD4 T cells. However, we noted in the preliminary experiments that IL-7-Tph cells were rarely induced from purified CD4 T cells and that the number of cells in a culture well affected the appearance of IL-7-Tph cells, suggesting the requirement of cell contact. In fact, we found that an addition of anti-HLA-DR antibody prevented the differentiation of IL-7-Tph cells, indicating an involvement of self-MHC recognition (Fig. 4A). Blocking costimulatory signals by CTLA4-Ig also inhibited the appearance of IL-7-Tph cells (Fig. 4B). The involvement of basal TCR-signaling in the development of IL-7-Tph cells, raising the possibility that the factors essential for the anti-TCR mAb-induced CXCL13-producing cell development [17], namely adding TGF-β and blocking IL-2, affect the development of IL-7-Tph cells. However, no difference was detected in the development of IL-7-Tph cells in the culture with and without TGF-β or anti-IL-2 mAb (Fig. 4C).

Based on the finding that the recognition of self-MHC molecules on antigen presenting cells was required for the development of IL-7-Tph cells, we purified naive or memory CD4 T cells by negative selection without using anti-CD4 mAb, which might interfere the interaction with self-MHC, and cultured them with non-T cell fraction of PBMC. Like the case of bulk PBMC culture, IL-7-Tph cells were induced from naive CD4 T cells (Fig. 4D). This also ensures that these are not derived from cTfh cells or cTph cells. Memory CD4 T cells also differentiated into Tph-like cells but less efficiently than naive CD4 T cells. Signals from self-MHC recognition and costimulation were required in both naïve and memory CD4 T cells for the development of IL-7-Tph cells (Fig. 4E).

### 3.4 Chemotaxis of IL-7Tph cells toward RASF

**Fig. 5:**
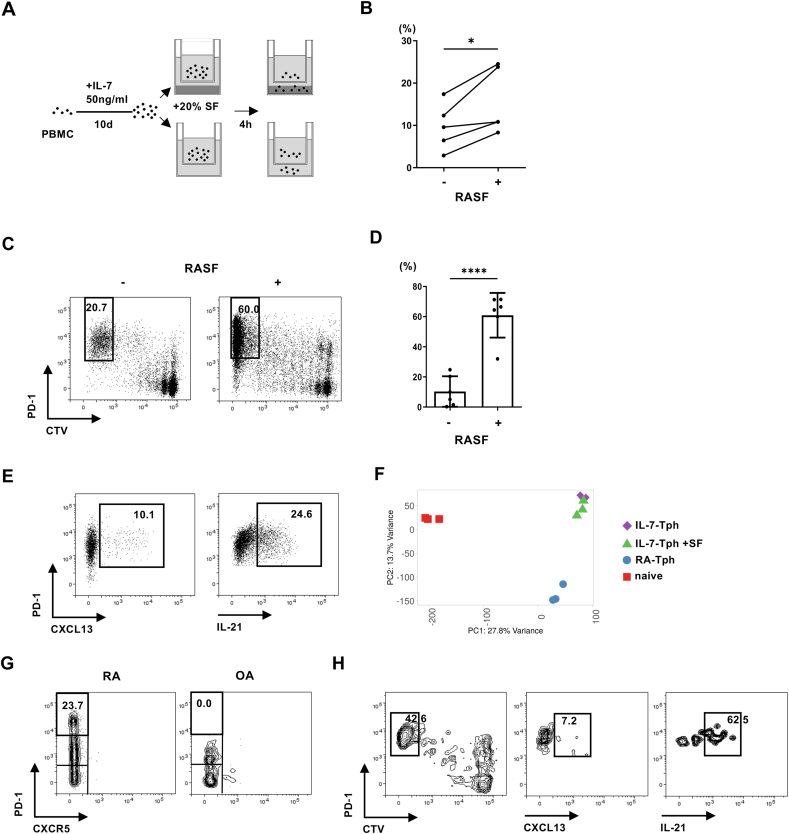
Multiple mechanisms account for the accumulation Tph cells in the joint of RA. (A) Experimental system for the chemotaxis assay. (B) The frequency of PD-1^high^ CTV^low^ IL-7-Tph cells migrated toward wells with or without RA SF (n = 5). (C) Representative dot plots showing the development of IL-7-Tph cells with or without RASF. (D) The frequency of PD-1^high^ CTV^low^ IL-7-Tph cells induced with or without RA SF (n = 6). (E) Representative dot plots showing the expression of CXCL13 and IL-21 in the IL-7-Tph cells induced with RASF after stimulation. (F) PCA analysis on the gene expression of IL-7-Tph cells with or without RA SF, RA-Tph, and naive CD4 T cells (n = 3). (G) Representative dot plots showing the presence and absence of PD-1^high^CXCR5- Tph cells in RA and OA joints, respectively. (H) Representative dot plots showing the development of IL-7-Tph cells from CD4 T cells in OA joints. Right panels show the expression of CXCL13 and IL-21 in PD-1^high^ CTV^low^ cells after stimulation.

Although the above experiments indicate homeostatic signals capable of inducing the development of Tph cells from PB CD4 T cells, Tph cells are much fewer in circulation compared to the joints, suggesting that Tph cells, once generated, migrate into the joints. To test this possibility, we performed chemotaxis assay. After inducting IL-7-Tph cells, PBMCs were placed in the upper wells of a Boyden chamber, and cells having migrated into the lower wells with or without RASF were harvested (Fig. 5A). We found that IL-7-Tph cells were enriched in the wells with RASF, suggesting preferential migration toward RA joints (Fig. 5B).

### 3.5 RASF promotes the development of IL-7-Tph cells

In addition to cellular influx, local differentiation of Tph cells in RA joints might contribute to the accumulation of Tph cells in RA joint. We therefore examined the effect of RASF during induction of IL-7-Tph cells (Fig. 5C and D). An addition of RASF greatly enhanced the generation of IL-7-Tph cells, but RASF alone rarely induced Tph-like cells in the absence of exogenous IL-7 (Supplementary Fig. 1). IL-7-Tph cells with RASF are qualitatively like those without RASF in that both produced CXCL13 spontaneously and IL-21 after stimulation (Fig. 5E). PCA analysis of bulk RNAseq confirmed their similarity, although IL-7-Tph cells with RASF positioned slightly closer to RA-Tph cells than those without RASF (Fig. 5F). So far, we have not identified the factors in RASF that promote the development of IL-7-Tph cells. Multiplex assay for a panel of cytokines and chemokines in RASF found no correlation with the frequency of IL-7-Tph cells induced by the RASF (Supplementary Fig. 2).

### 3.6 Induction of IL-7-Tph cells from CD4 T cells residing in non-inflammatory joint

To recapitulate the local differentiation of Tph cells in more physiological settings, we lastly examined whether joint-resident CD4 T cells in non-inflammatory joint give rise to IL-7-Tph cells. CD4 T cells in the synovium of OA, a degenerative joint disorder, were subjected for this experiment. Tph cells were not detected in OA CD4 T cells before culture, although the majority of which are memory-phenotype CD4 T cells in RA joint (Fig. 5G, data not shown). We found Tph-like cells, which expressed CXCL13 and IL-21, differentiated from OA CD4 T cells after culture with IL-7 (Fig. 5H). Thus, homeostatic signals and the inflammatory milieu of RA joint can induce Tph-like cells from resident CD4 T cells in an antigen-nonspecific manner.

## 4 Discussion

We demonstrated in this study that human CD4 T cells proliferated in response to homeostatic signals, including IL-7 and self-MHC recognition, and acquired phenotypes and functions compatible with Tph cells in RA joint. Such antigen-nonspecific mechanism of Tph cell development, which is further promoted by RASF, can explain the presence of a large number of Tph cells with diverse TCR repertoire in RA joints. Thus, the link between systemically dysregulated T cell homeostasis and local joint pathology of RA has been revealed. Similar mode of Tph cell development might occur in various inflammatory conditions.

Similar to our in vitro observation on human CD4 T cells, mouse CD4 T cells acquired Tph-like phenotype and functions after LIP in vivo [26]. Two modes of LIP exist for mouse CD4 T cells, fast and slow LIP, and the former is dependent on CD28-signaling and generates memory-phenotype T cells [30]. In fact, the Tph-like cells were detected in mouse CD4 T cells undergone fast LIP [26]. Consistent with this, we observed human CD4 T cells exhibiting fast proliferation and development of Tph-like cells in response to IL-7. Kiner also reported the expression of PD-1 on human CD4 T cells after culture with IL-7 [31]. On the other hand, a study showed that human naïve CD4 T cells cultured with autologous APC exhibit both fast and slow proliferation, but the former was independent of IL-7 [32]. The reason for the discrepancy is unclear, but we have observed fast proliferation of CD4 T cells without IL-7 in rare cases. Importantly, it was demonstrated that IL-7 enhanced TCR signalling in human T cells [33], through which IL-7 might induce the fast proliferation. In support of our hypothesis, an increased level of IL-7 in RA serum was reported [34], although there are conflicting reports [35,36]. It is also worth noting that IL-7-induced proliferation of CD4 T cells induces arthritis in a murine model of RA [37].

We demonstrated in this study similarities between the IL-7-Tph and RA-Tph cells. Yoshitomi et al. have reported the induction of CXCL13-producing Tph-like CD4 cells by a different culture system: culturing anti-CD3 mAb-stimulated naïve CD4 T cells in the presence of TGF-β, which is enhanced by blocking IL-2 [17]. There are substantial differences from our IL-7-Tph cells. A large portion of the IL-7-Tph and RA-Tph cells produced IL-21, but it was hardly produced by the CXCL13-producing cells [17]. Although they identified Sox4 as the critical transcription factor for CXCL13 production, transfecting Sox4 failed to induce the expression of IL-21 [18]. Our transcriptome analysis showed that Sox4 was not upregulated in RA-Tph cells or IL-7-Tph cells. We also observed blocking IL-2 did not promote the development of IL-7-Tph cells, in contrast to the case of CXCL13-producing cells. Despite such differences, both the CXCL13-producing cells and the IL-7-Tph cells spontaneously produced CXCL13, but not other cytokines, like RA-Tph cells. There might be unique regulatory mechanisms of CXCL13 production.

We found that only a portion of IL-7-stimulated CD4 cells showed fast proliferation and differentiated into Tph-like cells. Studies using TCR-transgenic mice speculated clonal competition as the limiting factor of LIP of naive T cells, which is affected by the affinity of TCR [[38], [39], [40]]. It is important to elucidate antigen specificity of the in vitro induced Tph-like cells, which might pave the way to understand human autoimmunity. Identification of the developing precursor cells in the culture is also essential for analysing intracellular signalling events in the development of IL-7-Tph cells, which might be elicited by the combination of homeostatic TCR, CD28, and IL-7 signalling and linked to cell proliferation. So far, it is known that IL-7-signalling suppressed the expression of BCL6, which is upregulated in Tfh but not Tph cells [41], suggesting distinct developmental pathway between Tph and Tfh cells. Our study also sheds light on an old observation that human T cells cultured with autologous antigen presenting cells exhibit spontaneous proliferation, the autologous mixed leukocyte reaction (AMLR) [42,43], which also depends on HLA-DR [44], but the antigen specificity of the AMLR T cells have not been clarified either.

The frequency of Tph cells in RA joint is much higher than the frequency of cTph in PB [8]. We showed in this study that IL-7-Tph cells showed chemotaxis toward RASF. The development of IL-7-Tph cells was greatly promoted by adding RASF to the culture. Importantly, IL-7-Tph cells can also be induced from CD4 T cells residing in non-inflamed joints. These likely occur because an increased level of IL-7 protein in RASF and IL-7 mRNA in RA fibroblasts was reported [[45], [46], [47]]. Although we hardly detected IL-7 in our RASF samples (Supplemental Fig. 2), and RASF alone failed to induce Tph-like cells (Supplemental Fig. 1), T cells might receive IL-7 signal in the synovial membrane where they can contact with antigen-presenting cells, but not in SF. Thus, there might be two pathways of antigen-nonspecific Tph cell accumulation in RA joint, infiltration of Tph cells from the circulation and local development of Tph cells. In fact, Argyriou et al. demonstrated clonal relationships between Tph and other CD4 T cell subsets in RA joint [10], suggesting conversion of Tph cells from other memory CD4 T cells residing in the joint.

Given that Tph cells are implicated in the formation of ELOs, our results propose a novel mechanism of local ELO development. Thus, chronic inflammation induces antigen-nonspecific development of Tph cells, which spontaneously produce CXCL13, recruiting B cells expressing CXCR5. Interestingly, gene expression analysis suggested an involvement of IL-7 signaling pathway in ELO formation in RA synovium [48]. Our findings are also supported by the observation that Tph cells as well as ELO formation in RA synovium decrease after treatment [49]. Cessation of inflammation disturbs the maintenance of ELO, suggesting that cytokine-driven rather than antigen-driven reaction induces ELO formation. This might apply to other inflammatory disorders with ELO formation, such as pSS and thyroiditis. Thus, our results speculate the presence of antigen-nonspecific T cell-mediated immunopathogenesis in chronically inflamed tissues.

## CRediT authorship contribution statement

**Ryosuke Tsurui:** Writing – review & editing, Writing – original draft, Investigation, Formal analysis, Data curation, Conceptualization. **Hisakata Yamada:** Writing – review & editing, Writing – original draft, Investigation, Formal analysis, Data curation, Conceptualization. **Takahiro Natori:** Writing – review & editing, Investigation, Formal analysis, Data curation. **Motoki Yoshimura:** Writing – review & editing, Investigation. **Yukio Akasaki:** Writing – review & editing, Data curation. **Shinya Kawahara:** Writing – review & editing, Data curation. **Hiroaki Niiro:** Writing – review & editing, Supervision, Data curation. **Yuya Kunisaki:** Writing – review & editing, Supervision, Formal analysis. **Yasuharu Nakashima:** Writing – review & editing, Supervision.

## Funding

This work was supported in part by The Clinical Research Foundation, Fukuoka, Japan.

## Declaration of competing interest

The authors declare the following financial interests/personal relationships which may be considered as potential competing interests:Hiroaki Niiro reports a relationship with Bristol-Myers Squibb that includes: speaking and lecture fees. If there are other authors, they declare that they have no known competing financial interests or personal relationships that could have appeared to influence the work reported in this paper.

## Data Availability

Data are available in the article itself and its supplemental materials. The raw RNAseq data have been deposited to GEO (GSE273760).
