# Supplementary material for: Homeostatic signals, including IL-7 and self-MHC recognition, induce the development of peripheral helper T cells, which are enriched in the joints of rheumatoid arthritis
Source: J Transl Autoimmun. 2024 Oct 30;9:100258. doi: 10.1016/j.jtauto.2024.100258 (PMC11567946; doi:10.1016/j.jtauto.2024.100258)
Supplement: Multimedia component 1 [file mmc1.pdf]

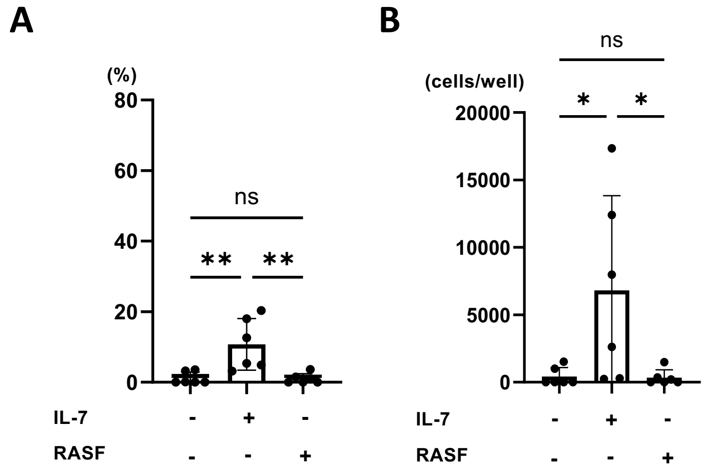

Supplemental figure 1

Development of Tph-like cells after culture either with IL-7 or RASF. (A) The frequency (A) or number (B) of PD-1^{high} CTV^{low} Tph-like cells induced in culture conditions as indicated below is shown (n=6). *$p < 0.05$, **$p < 0.01$.
