# Supplementary material for: Homeostatic signals, including IL-7 and self-MHC recognition, induce the development of peripheral helper T cells, which are enriched in the joints of rheumatoid arthritis
Source: J Transl Autoimmun. 2024 Oct 30;9:100258. doi: 10.1016/j.jtauto.2024.100258 (PMC11567946; doi:10.1016/j.jtauto.2024.100258)
Supplement: Multimedia component 2 [file mmc2.pdf]

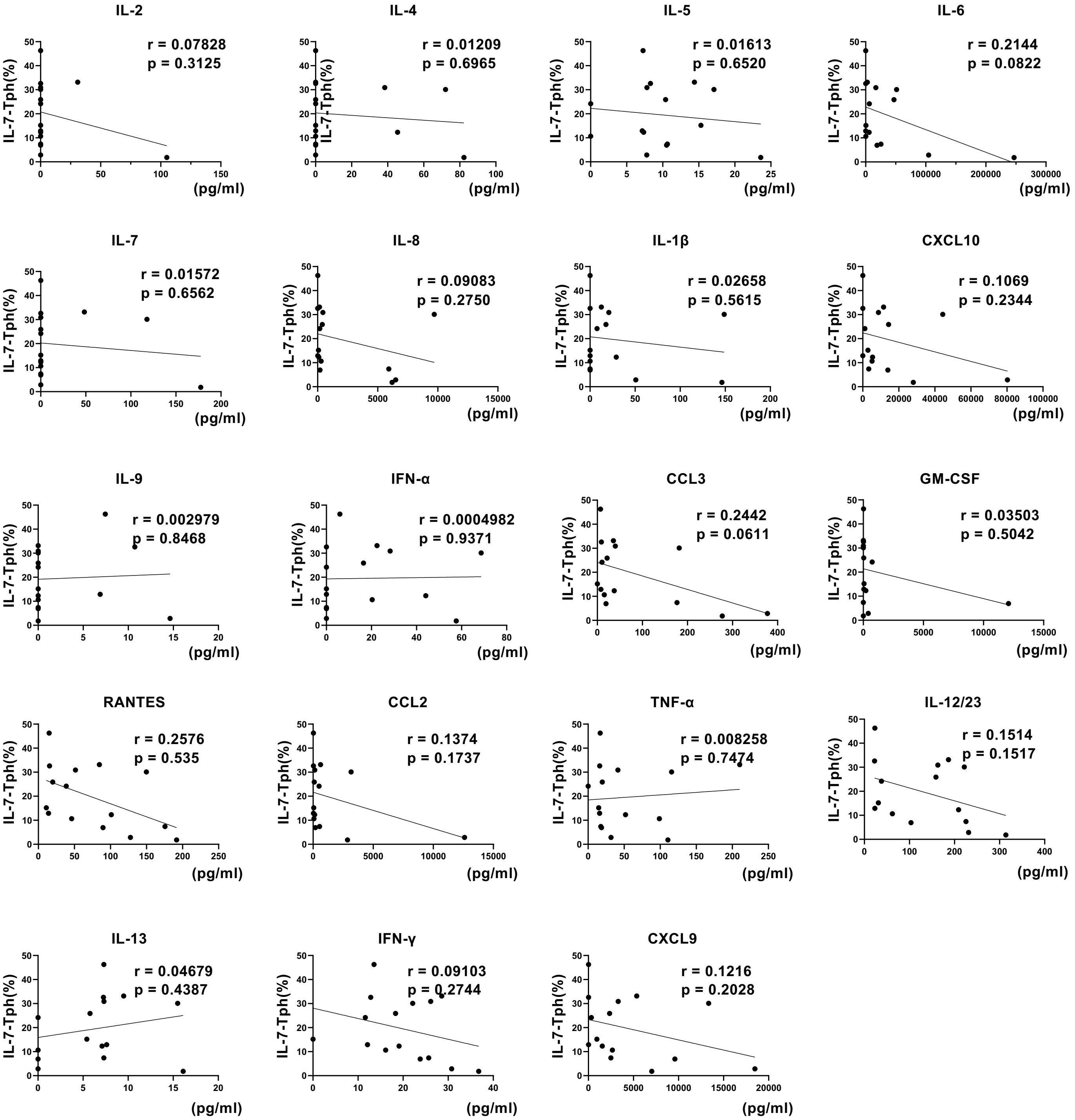

Supplemental figure 2

Correlation between the concentration of the frequency of IL-7-Tph cells and the concentration of cytokines and chemokines in RASF added to the culture (n=15). The cytokines and chemokines were detected by a multiplex assay. The result of Pearson correlation coefficient analysis is indicated in each panel.
