# Supplementary material for: Homeostatic signals, including IL-7 and self-MHC recognition, induce the development of peripheral helper T cells, which are enriched in the joints of rheumatoid arthritis
Source: J Transl Autoimmun. 2024 Oct 30;9:100258. doi: 10.1016/j.jtauto.2024.100258 (PMC11567946; doi:10.1016/j.jtauto.2024.100258)
Supplement: Multimedia component 3 [file mmc3.pdf]

Supplemental table 1.

	RA (SF, n=18)	RA (PBMC, n=6)	OA (n=3)
Age (years)	62 ± 8.2	65.5 ± 10.5	58 ± 7.8
Sex (female/male)	16/2	5/1	2/1
RF/ACPA positive (%)	100	100	n.a.
RA duration (years)	9.5±8.3	14.5±9.6	n.a.
CRP (mg/L)	11±13.7	1±7.6	0.4±0.3
Methotrexate (%)	75	0	-
Biological DMARDs (%)	44	50	-
Glucocorticoids (%)	38	33	-
Kellgren-Lawrence grade	n.a.	n.a.	grade 4, 2<br>grade 3, 1

RA, rheumatoid arthritis; SF, synovial fluid; PBMC, peripheral blood mononuclear cells; OA, osteoarthritis; RF, rheumatoid factor; ACPA, anti-citrullinated protein/peptide antibody; CRP, C-reactive protein; DMARD, disease modified anti rheumatic drug.
