# Supplementary material for: Homeostatic signals, including IL-7 and self-MHC recognition, induce the development of peripheral helper T cells, which are enriched in the joints of rheumatoid arthritis
Source: J Transl Autoimmun. 2024 Oct 30;9:100258. doi: 10.1016/j.jtauto.2024.100258 (PMC11567946; doi:10.1016/j.jtauto.2024.100258)
Supplement: Multimedia component 4 [file mmc4.pdf]

supplemental table 2.

Antibodies	Source	Identifier
Alexa Fluor 488 anti-human CXCR5 (RF8B2)	BD Bioscience	558112
PE anti-human 45RA (HI100)	BioLegend	304107
PE-Cy7 anti-human PD-1 (J105)	Thermo Fisher Scientific	25-2799-42
APC anti-human CD4 (RPA-T4)	BioLegend	300514
APC-H7 anti-human CD4 (RPA-T4)	BD Bioscience	560251
FITC anti-human HLA-DR (G46-6)	BD Bioscience	560944
PE anti-human CD69 (FN50)	Thermo Fisher Scientific	12-0699-41
Alexa Fluor 647 anti-human ICOS (C398.4A)	BioLegend	313515
FITC anti-human CCR6 (R6H1)	Thermo Fisher Scientific	11-1969-41
PE anti-human CXCR3 (1C6)	BD Bioscience	560928
APC anti-human CXCR5 (J252D4)	BioLegend	356907
BV450 anti-human CD3 (UCHT1)	BD Bioscience	560366
BV510 anti-human 45RA (HI100)	BioLegend	304142
Alexa Fluor 488 anti-human IL4 (8D4-8)	Thermo Fisher Scientific	53-7049-41
PE anti-human TNFα (MAb11)	Thermo Fisher Scientific	12-7349-71
PerCP anti-human GM-CSF (BVD2-21C11)	BioLegend	502311
APC anti-human IL-2 (MQ1-17H12)	BD Bioscience	561054
Alexa Fluor 488 anti-human IL-17a (N49-653)	BD Bioscience	560489
PE-Cy7 anti-human IL-21 (3A3-N2)	Thermo Fisher Scientific	12-7219-41
PerCP anti-human INF-γ (4S.B3)	BioLegend	502525
APC anti-human CXCL13 (5334)	R&D Systems	MAB202-SP
FITC anti-human CD27 (M-T271)	BD Bioscience	560986
PE anti-human 45RO (UCHL1)	Thermo Fisher Scientific	12-0457-41
APC anti-human CD19 (HIB19)	Thermo Fisher Scientific	17-0199-42
BV450 anti-human CD38 (HIT2)	Tonbo Bioscience	75-0389-T025
Biotin anti-human CXCR5 (MU5UBEE)	Thermo Fisher Scientific	13-9185-82
Biotin anti-human PD-1 (J105)	Thermo Fisher Scientific	13-2799-80
PE Streptavidin	BioLegend	405204
